# A neuroendocrine carcinoma with a well-differentiated adenocarcinoma component arising in Barrett’s esophagus: a case report and literature review

**DOI:** 10.1186/s40792-018-0511-7

**Published:** 2018-08-29

**Authors:** Shunsuke Doi, Sohei Matsumoto, Kohei Wakatsuki, Kazuhiro Migita, Masahiro Ito, Tomohiro Kunishige, Hiroshi Nakade, Kinta Hatakeyama, Chiho Ohbayashi, Masayuki Sho

**Affiliations:** 1 Department of Surgery, Nara Medical University, 840 Shijo-cho, Kashihara, Nara 634-8522 Japan; 2 Department of Diagnostic Pathology, Nara Medical University, 840 Shijo-cho,, Kashihara, Nara 634-8522 Japan

**Keywords:** Esophagus, Neuroendocrine carcinoma, Adenocarcinoma, Barrett’s esophagus

## Abstract

**Background:**

An esophageal neuroendocrine carcinoma arising in Barrett’s esophagus is extremely rare. Here, we report a case of an esophageal neuroendocrine carcinoma with a well-differentiated adenocarcinoma component arising in Barrett’s esophagus and review the literature.

**Case presentation:**

A 71-year-old man with no symptoms was admitted to our hospital because of the detection of an esophagogastric junction tumor on regular upper endoscopy screening. Endoscopy revealed a sliding hiatal hernia and an approximately 10 mm elevated mass at the esophagogastric junction. Biopsy showed a moderately differentiated tubular adenocarcinoma. Computed tomography did not indicate lymph node metastasis or distant metastasis. Proximal gastrectomy with D1 lymph node dissection was performed along with jejunal interposition. On immunohistochemical staining, the tumor was positive for chromogranin A and synaptophysin. Ki-67 was positive in 40% of the tumor cells. The histological diagnosis was a neuroendocrine carcinoma with a well-differentiated adenocarcinoma component arising in Barrett’s esophagus. The postoperative course was good, and the patient was discharged on the twentieth postoperative day. He has remained free of the disease at 36 months postoperatively.

**Conclusions:**

Barrett’s esophagus may be related to the development of a neuroendocrine carcinoma.

## Background

An esophageal neuroendocrine carcinoma (NEC) is relatively rare and accounts for 0.4–5.9% of all esophageal carcinomas [1–3]. It is known to show aggressive progression, poor survival outcomes, and resistance to chemoradiation therapy [4]. Barrett’s esophagus (BE) is a well-known premalignant condition associated with the occurrence of an esophageal adenocarcinoma [5]. An esophageal NEC arising in BE is extremely rare. Therefore, its clinicopathological and immunohistochemical features are not well understood. Moreover, its diagnosis and treatment remain clinically challenging. Herein, we report a case of an esophageal NEC arising in BE and review the literature.

## Case presentation

**Fig. 1:**
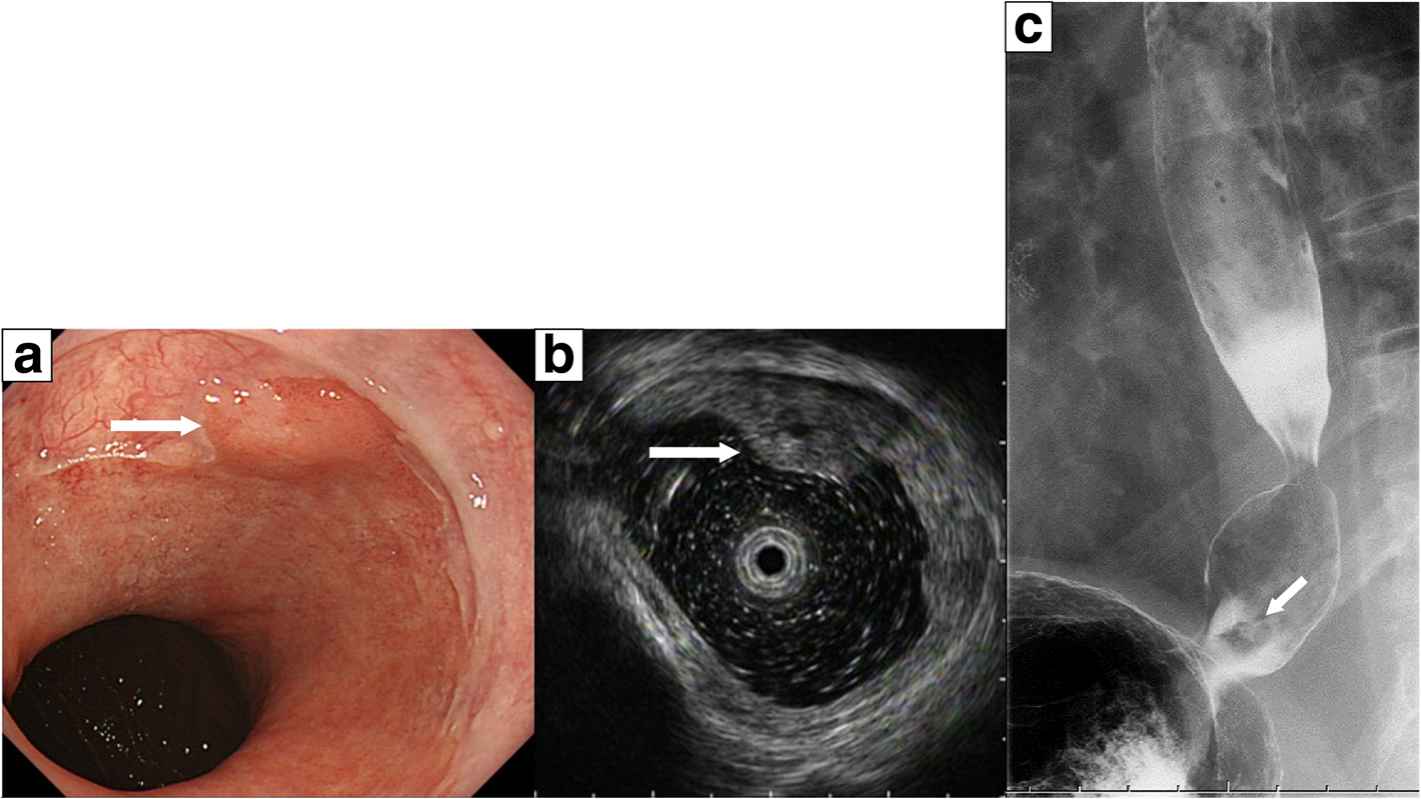
Images obtained before surgery. **a** Endoscopy shows a sliding hiatal hernia and an approximately 10 mm elevated mass at the esophagogastric junction. **b** Endoscopic ultrasonography shows a mass having mixed echogenicity in the esophageal wall, with partial invasion of the submucosal layer. **c** Upper gastrointestinal imaging shows an elevated lesion at the esophagogastric junction

A 71-year-old man was admitted to our hospital because of the detection of an esophagogastric (EG) junction tumor on regular upper endoscopy screening. He had no symptoms, such as dysphagia, epigastric fullness, and gastroesophageal reflux. His medical history included hepatolithiasis, and he had undergone hepatic left lateral segmentectomy at 50 years of age. Physical examination showed no remarkable findings, and laboratory examinations, including assessment of serum tumor markers, such as carcinoembryonic antigen and carbohydrate antigen 19-9, were normal. Endoscopy revealed a sliding hiatal hernia and an approximately 10 mm elevated mass at the EG junction (Fig. 1a). Endoscopic ultrasonography showed a mass having mixed echogenicity in the esophageal wall, with partial invasion of the submucosal layer (Fig. 1b). Upper gastrointestinal imaging showed an elevated lesion at the EG junction (Fig. 1c). A biopsy specimen was obtained, and the pathological diagnosis on analysis of the specimen was a differentiated tubular adenocarcinoma. Computed tomography did not indicate lymph node metastasis or distant metastasis. The clinical diagnosis was esophageal cancer (cT1bN0M0 cStage I according to the eighth edition of the Union for International Cancer Control classification) [6]. Proximal gastrectomy with D1 lymph node dissection was performed along with jejunal interposition.

**Fig. 2:**
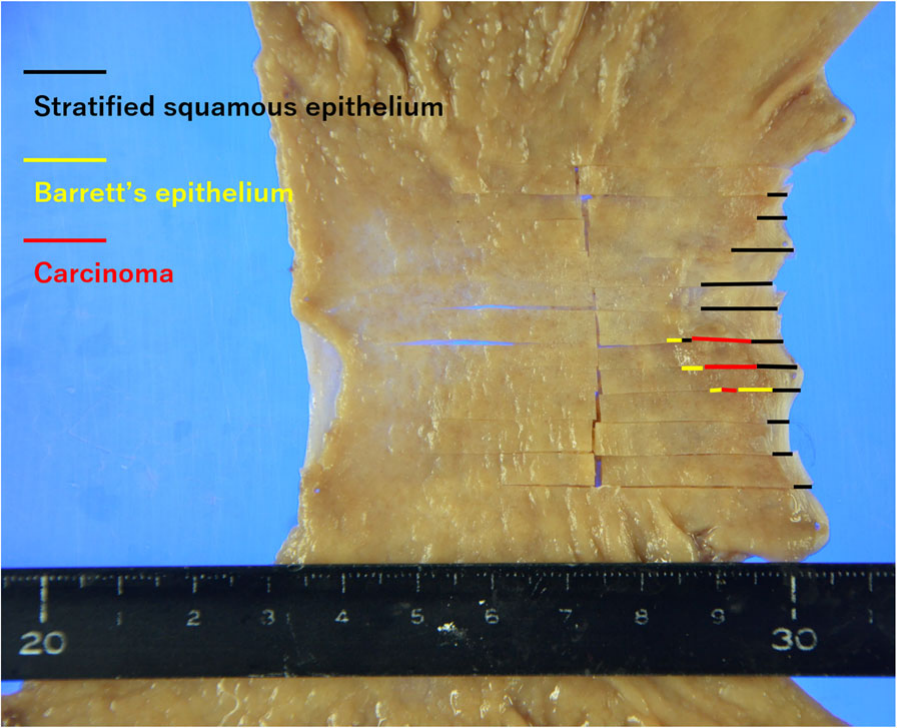
Macroscopic findings. The surgical specimen shows an elevated mass (type 0-IIa; 10 × 8 mm) at the esophagogastric junction. Stratified squamous epithelium, Barrett’s epithelium, and the carcinoma are indicated

**Fig. 3:**
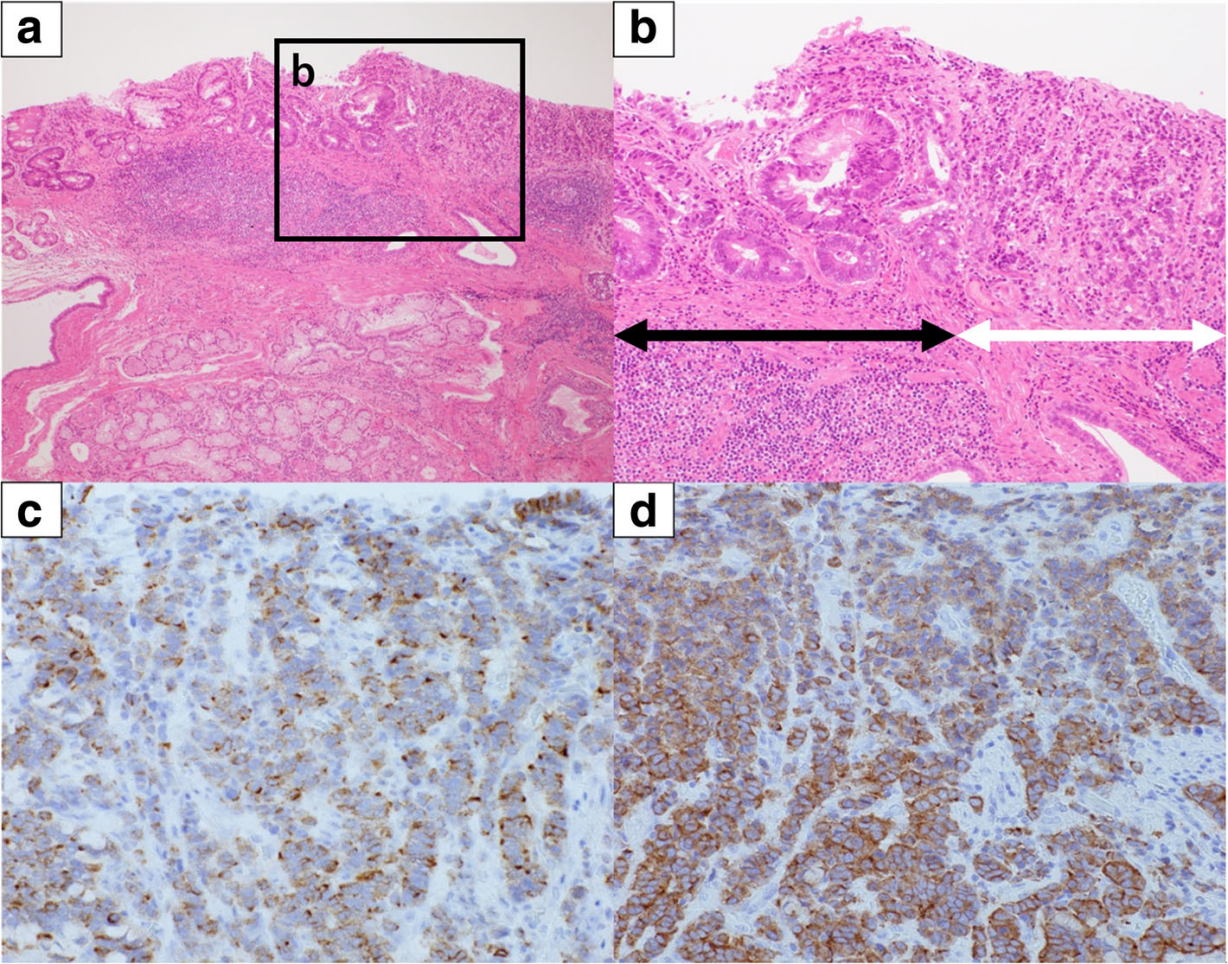
Histopathological findings. **a** Hematoxylin-eosin staining shows that the tumor arose in Barrett’s epithelium (× 40). **b** Magnification of the part indicated by the *square* in (**a**) (× 100). The tumor shows two components (*black* and *white arrows*). The *black arrow* indicates a well-differentiated adenocarcinoma, whereas the *white arrow* indicates a neuroendocrine carcinoma. **c** Immunohistochemical staining for chromogranin A is positive (× 200). **d** Immunohistochemical staining for synaptophysin is positive (× 200)

Macroscopically, the surgical specimen showed an elevated mass (10 × 8 mm) in the EG junction (Fig. 2). Microscopic examination revealed a carcinoma associated with BE. The carcinoma, Barrett’s epithelium, and stratified squamous epithelium are indicated in Fig. 2. Hematoxylin-eosin staining showed that the tumor was composed of small-to-intermediate cells with scant cytoplasm and irregular hyperchromatic nuclei and was growing with nuclear palisading and tubular structures. A well-differentiated adenocarcinoma component was present independently. The neoplasm arose in Barrett’s epithelium (Fig. 3a and b). Infiltration of the submucosal layer to a depth of < 200 μm was noted. Lymphovascular invasion was not identified. The margins of the specimen were free of tumor cells. On immunohistochemical staining, the tumor was positive for chromogranin A and synaptophysin (Fig. 3c and d). Ki-67 was positive in 40% of the tumor cells. Thus, the histological diagnosis was an NEC with a well-differentiated adenocarcinoma component arising in BE. No metastasis in the lymph nodes was noted on histological examination. The pathological diagnosis was esophageal cancer (pT1bN0M0 pStage I). The resection margins were free of tumor cells (R0 resection). The patient’s postoperative course was good, and he was discharged on the twentieth postoperative day. He has remained free of the disease at 36 months postoperatively.

## Discussion

The World Health Organization’s 2010 cancer classification considered NECs as a subgroup of neuroendocrine neoplasms [7]. In brief, neuroendocrine neoplasms can be categorized into three grade-based groups, and among them, high-grade neuroendocrine neoplasms are NECs. They are defined as neoplasms having > 20 mitotic figures/10 high-power fields or a Ki67 index of > 20%. Esophageal NECs are known to be rare and aggressive neoplasms [1–4]. BE has been recognized as a precancerous lesion with high carcinogenic potential, and special columnar epithelium has been considered as a carcinogenic site. Cases of esophageal adenocarcinoma and squamous cell carcinoma (SCC) arising in BE have been reported and are relatively well researched [5, 8, 9]. However, an esophageal NEC arising in BE is extremely rare. To search the literature for such cases, we used keywords such as “neuroendocrine carcinoma” and “Barrett’s esophagus.” Moreover, we retrieved cases from the relevant reference lists. We found only eight reports of an esophageal NEC arising in BE [10–17] (Table 1).

**Table 1:** Summary of cases of neuroendocrine carcinomas arising in Barrett’s esophagus

No.	Author	Age (y)	Sex	Chief complaints	Reflux symptoms	Siewert classification	Tumor size (mm)	*T*	*N*	*M*	Stage	Tumor type	Chromogranin	Synaptophysin	Treatment	Postoperative survival
1	Slavin (1994)	63	F	Dysphagia and weight loss	(+)	Type 2	50	1	1	1	IV	Pure NEC	(+)	(−)	Esophagogastrectomy	Died at 3 months
2	Saw (1997)	43	F	Epigastric pain	(+)	Type 1	25	N.A.	N.A.	N.A.	N.A.	NEC with adenocarcinoma	(+)	(+)	Esophagectomy	Alive at 36 months
3	Saint Martin (1999)	54	F	Dysphagia	(+)	Type 2	N.A.	N.A.	N.A.	N.A.	N.A.	Pure NEC	(−)	(+)	Esophagogastrectomy	N.A.
4	Chen (2000)	64	M	Dysphagia	(−)	Type 1	40	N.A.	N.A.	N.A.	N.A.	Pure NEC	N.A.	N.A.	CRT	Alive at 14 months
5	Wilson (2000)	51	M	Epigastric discomfort and dysphagia	(+)	Type 2	40	1b	0	0	IA	NEC with adenocarcinoma	(−)	(+)	Esophagogastrectomy	N.A.
6	Gonzalez (2003)	75	M	Anorexia, weight loss, and dysphagia	(+)	Type 2	45	2	0	0	IB	NEC with adenocarcinoma	(−)	(+)	Esophagogastrectomy	N.A.
7	Bibeau (2008)	54	M	Epigastric discomfort	(+)	Type 2	20	2	0	0	IB	NEC with adenocarcinoma	(+)	(+)	CRT and esophagectomy	Alive at 72 months
8	Markogiannakis (2008)	62	M	Epigastric pain, epigastric fullness, dysphagia, anorexia, and weight loss	(+)	Type 2	25	2	1	0	IIB	Pure NEC	(+)	(−)	CRT and esophagogastrectomy	N.A.
9	Current report	71	M	No symptoms	(−)	Type 2	10	1b	0	0	IA	NEC with adenocarcinoma	(+)	(+)	Proximal gastrectomy	Alive at 36 months

*N.A.* not available, *NEC* neuroendocrine carcinoma, *CRT* chemoradiotherapy

Among patients with esophageal NECs, the most common symptoms are dysphagia, anorexia, and weight loss. These symptoms are similar to those of advanced-stage esophageal cancer. Esophageal NECs are often diagnosed in the advanced stage. Consequently, the prognosis of patients with esophageal NECs is dismal, and the median overall survival duration is 14–28.5 months [1–4]. On the other hand, patients with esophageal NECs arising in BE have reflux symptoms, which might be associated with BE itself (Table 1). Considering the reasons for these differences, esophageal NECs arising in BE might be detected in the early stage and might show better patient survival. In our assessment of the eight previous cases of esophageal NECs arising in BE and our case (nine total cases), we found that seven cases had been identified with T1 or T2 tumor depth. Furthermore, half of the cases were identified at stage I. Thus, most patients with esophageal NECs arising in BE showed long-term survival. However, one patient died relatively early from a cerebral infarction. Our patient was asymptomatic, and the tumor size was the smallest among all the cases of esophageal NECs arising in BE. The patient was alive without recurrence at 36 months postoperatively. Therefore, careful endoscopic surveillance is important for early detection of Barrett’s-associated cancers.

In our literature review, we found that most patients with esophageal NECs arising in BE underwent esophagogastrectomy. The optimal surgical procedure and reconstruction approach for cancer at the EG junction are controversial. We have previously reported that metastasis to lymph node stations 4d, 5, and 6 is rare in cancer located at the EG junction [18, 19]. Additionally, a nation-wide retrospective study in Japan showed that lymphadenectomy for stations 4, 5, and 6 is not recommended for an adenocarcinoma at the EG junction [20]. Furthermore, the JCOG 9502 study recommended an abdominal approach for EG junction cancers with esophageal invasion of 3 cm or less [21]. Lower mediastinal node dissection might contribute in improving the survival of patients with EG junction cancer. However, due to low dissection rates for nodes of the middle and upper mediastinum, no conclusive result was obtained regarding the optimal extent of node dissection in this region [22]. Therefore, we selected proximal gastrectomy with jejunal interposition in the present patient. Jejunal interposition is effective for preventing reflux esophagitis after proximal gastrectomy [23, 24]. However, further research is needed to obtain better short- and long-term outcomes in patients with NECs at the EG junction.

Pathologically, the relationship between an esophageal NEC and BE has not been clarified. Among the nine cases of esophageal NECs arising in BE, five had an adenocarcinoma component. On the other hand, esophageal NECs with an SCC component have been presented in several previous reports [25–29]. However, Ho et al. has proposed that multipotent neoplastic stem cells are common precursors for adenocarcinoma, SCC, and NEC of the esophagus [25]. Under carcinogenic stimulation, such as that in BE, the multipotent cells are activated and transformed into various malignant cells.

## Conclusion

An esophageal NEC arising in BE is extremely rare. BE may be related to the development of NECs. Further studies are required to clarify the mechanisms of this disease.

## Abbreviations

BE: Barrett’s esophagus
EG: Esophagogastric
NEC: Neuroendocrine carcinoma
SCC: Squamous cell carcinoma
